# Optimized performance III-nitride-perovskite-based heterojunction photodetector *via* asymmetric electrode configuration†

**DOI:** 10.1039/c9ra08823g

**Published:** 2020-02-11

**Authors:** Somak Mitra, Mufasila Mumthaz Muhammed, Norah Alwadai, Dhaifallah R. Almalawi, Bin Xin, Yusin Pak, Iman S. Roqan

**Affiliations:** King Abdullah University of Science and Technology (KAUST), Physical Sciences and Engineering Division Thuwal 23955-6900 Saudi Arabia iman.roqan@kaust.edu.sa; Department of Physics, Princess Nourah Bint Abdulrahman University (PNU) Riyadh 11671 Saudi Arabia

## Abstract

Organometal halide perovskite photodetectors have recently drawn significant attention due to their excellent potential to perform as broadband photodetectors. However, the photoresponse in the ultraviolet (UV) spectrum can be improved by introducing wide bandgap semiconductors. In this work, we report on a methylammonium lead iodide/p-type gallium nitride (MAPI/p-GaN) heterojunction photodetector. We demonstrate that the device is capable of detecting in the UV region by p-GaN being hybridized with MAPI. We further investigate different symmetric and asymmetric metal-electrode contacts to enhance the device performance including the response time. The asymmetric electrode configuration is found to be the most optimal configuration which results in high photoresponse (photo-responsivity is 55 mA W^−1^ and fall time < 80 ms). As the light illumination occurs through the GaN side, its presence ultimately reduces MAPI degradation due to efficient absorption of the UV photons by GaN film.

## Abstract 2

Enhanced perovskite/GaN-based broad-band photodetector is demonstrated by optimizing electrode configurations. The detection capability of the optimized perovskite/GaN structure was extended to UV range with fast response and high responsivity.

## Graphical abstract

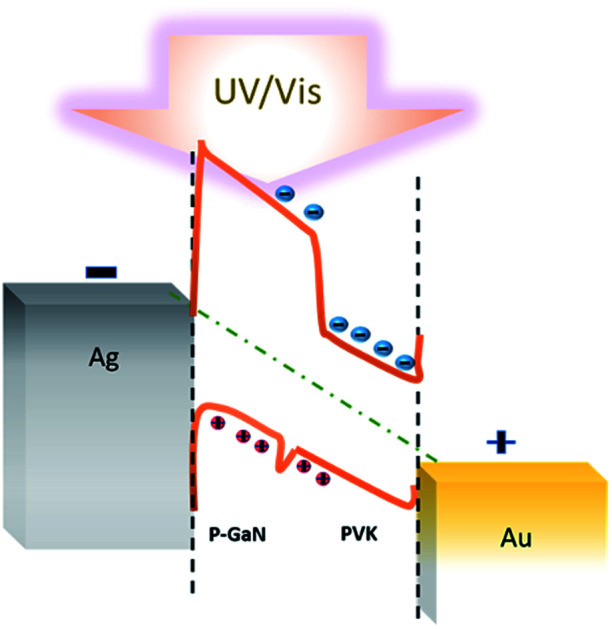

## Introduction

Organometallic halide perovskites in the form of CH_3_NH_3_PbX_3_ (where X denotes a halide) (PVK-MAPI), have shown excellent performance in a broad absorption range.^1,2^ Their excellent photoelectric conversion rate has prompted perovskite use in different optoelectronic applications. Recently, broadband photodetectors (PDs) based on PVK have gained considerable attention, as a very thin layer of perovskite can absorb the incident light as a result of the large absorption coefficient and high carrier mobility.^3^ Thus, organic–inorganic halide perovskite materials are promising candidates for PDs and photovoltaic devices.^4,5^ Moreover, there is a growing need for broad-spectrum PDs (covering UV, visible and IR range), which should be stable at room temperature as well as economically viable to ensure their broad applicability in different fields, such as environmental and biological sensors, video imaging and optical communications.^6–8^ Although, it is well known that MAPI is characterized by highly efficient photogeneration in the visible spectrum, the high-energy photons in the UV part of the spectrum cannot be accessed efficiently, as the narrow bandgap of PVK does not allow the material to operate efficiently in the UV regime.^8–10^ To resolve this issue, wide bandgap semiconductor/PVK-based heterojunction devices were fabricated. In such structures, wide bandgap materials can convert the high-energy photons in UV region to photocurrent,^11^ while the visible photons can be converted by PVK. Thus, several researchers have attempted to obtain ZnO/MAPI-based heterojunction PDs.^12–17^ However, in each of these cases, rapid material degradation was observed due to interaction with oxygen in the atmospheric moisture and metal oxide materials,^18^ and direct device exposure to UV light.^19^ Thus, obtaining stable and durable PVK devices is still challenging. There is a demand for improving heterojunction PDs incorporating PVK, including their stability and durability, as well as their photoresponse.

To meet these aims, an alternative wide bandgap material that can be used to access the high-energy photons is required for PVK-based applications. GaN is the best candidate for this purpose, as it is highly stable and is sensitive to UV light due to its wide and direct bandgap.^20,21^ Therefore, PVK-nitride based heterojunction devices should be considered for PD applications. It is well known that the band alignment between metal contacts and active layers can affect the device performance. However, the role of different contact configurations in optimizing the device performance, including the photoresponse speed, has not been studied. Thus, addressing this gap in the extant knowledge is essential for obtaining high-performance PVK/semiconductor-based PDs.

Here, we report on a heterojunction structure of CH_3_NH_3_PbI_3_ MAPI/p-type GaN PD, which permits access to the broadband spectrum and generates photoelectrons efficiently. The functionality of MAPI-based PDs is extended to the UV spectral region due to the wide GaN bandgap. We show that the electrode configuration allows maximum amount of photoelectrons to be collected and improves the PD characteristics. Moreover, our MAPI/p-GaN-based PD structure does not allow direct interaction of UV radiation with the MAPI layer, thus preventing material degradation.

## Experimental section

A commercial p-GaN film grown on a (0001) sapphire substrate (Cermet, Inc., USA) was used. The p-GaN/sapphire film was doped with Mg (∼1 × 10^20^ cm^−3^) as an acceptor dopant. First, p-GaN films were cleaned with deionized water, followed by ultrasonication in acetone and isopropanol for 5 min each, and a final cleaning step is performed in deionized water flow. To prepare the MAPI solution, the perovskite powder of >99% purity (Xi'an Polymer Light Technology Corp.) (309.95 mg) was dissolved in 1 mL of *N*,*N*-dimethylsulfoxide (DMF) solvent to make a solution. Then, the solution was stirred on a hot plate at 70 °C until fully dissolved. The p-GaN samples were heated on a hot plate at 100 °C for 30 min. Next, MAPI layer (CH_3_NH_3_PbI_3_) was deposited on the p-GaN films by a simple spray-coating method, using an airbrush (pro series BD-132), by employing nitrogen as a carrier gas under constant pressure. The substrate temperature was maintained at 60 °C. The MAPI/p-GaN samples were transferred directly to a high vacuum electron-beam (e-beam) evaporation deposition chamber to fabricate the Au and Ag contacts, using slow rate deposition.

Structural characterizations of the samples were performed by scanning electron microscopy (SEM), using FEI Nova Nano 630 system, while maintaining the accelerating voltage at 5 kV. X-ray diffraction (XRD) was carried out by employing D8 XRD Discover with Cu Kα radiation (*λ* = 1.5406 Å) at 40 kV (40 mA). To investigate the absorption properties, steady-state absorption measurements were performed by Agilent Cary 5000 UV-VIS-NIR spectrometer. The room temperature (RT) photoluminescence (PL) spectra of the p-GaN/PVK samples were examined by using He–Cd laser (325 nm) attached to the Horiba Aramis Jobin Yvon micro-PL system. The focused laser power was 5 mW and the grating was 600 g mm^−1^. The spectra were collected by Andor monochromator attached to a charge-coupled device camera. The PD photoresponse was investigated by conducting current–voltage (*I*–*V*) and current–time (*I*–*T*) measurements using a Keithley 2400 source meter. A halogen lamp with tunable power attached to an electro-mechanical shutter system (Thorlab SH05) served as a light source. Kelvin probe measurements were performed to calculate the work fucntions of p-GaN and MAPI films.

## Results and discussion

**Fig. 1:**
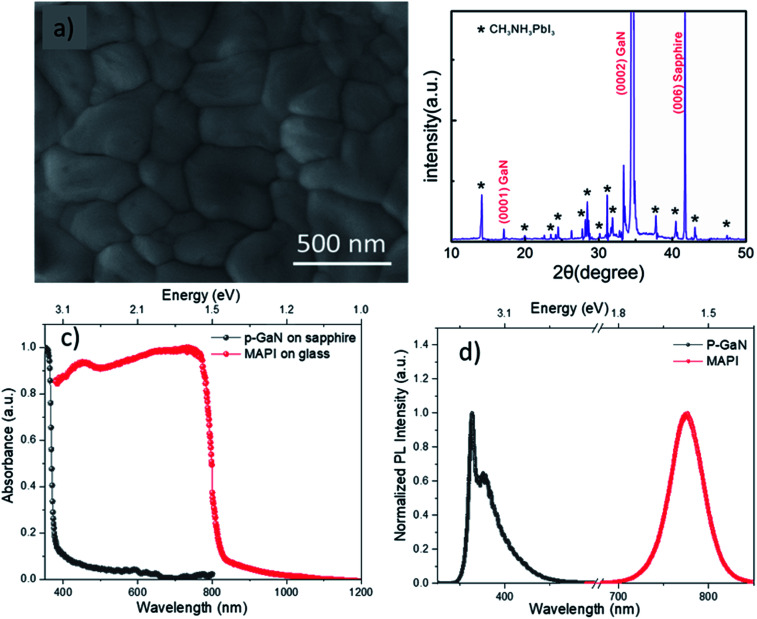
(a) SEM image and (b) XRD spectrum of the MAPI layer deposited on p-GaN film. (c) The absorption measurements of the MAPI film on a glass and the p-GaN film on sapphire substrate; (d) PL spectra of the p-GaN thin film and the MAPI film.

Fig. 1a shows an SEM image of the spray-coated MAPI on p-GaN film, which is characterized by a compact and highly smoother surface morphology with a larger crystal grain size compared to that synthesized on metal oxide.^17^ Moreover, the surface is shown to be pinhole free at the grain edges. These large pinhole-free grains help reduce the grain-boundary energy and favor charge carrier transportation.^1^ Thus, the SEM measurements demonstrate a continuous homogeneous MAPI film of a good crystalline quality covered GaN film completely. The cross-sectional image of the MAPI spray-coated film is shown in the ESI (Fig. S1†). The image pertains to an identical device prepared under the same conditions, revealing a MAPI film thickness of around 2 μm.

The crystallinity of the MAPI/p-GaN structure was analyzed *via* XRD measurements. The *θ*–2*θ* spectrum shown in Fig. 1b confirms that a strong GaN (0002) reflection peak emerges at 34.6°, indicating a single crystal GaN film is grown along (0001) direction. Moreover, a sapphire substrate peak is observed at 41.9°, corresponding to the (0001) substrate growth direction. For MAPI layer, we observed the emergence of a series of diffraction peaks that are in good agreement with the published data and the theoretically calculated values related to the tetragonal phase of CH_3_NH_3_PbI_3_ PVK.^24^ Typical MAPI peaks were observed at 14.1°, 19.9°, 23.5°, 24.4°, 28.1°, 28.4°, 30.9°, 31.6°, 32.8°, 37.2°, 40.4°, 43.0°, and 47.6°, corresponding to (110), (200), (211), (202), (004), (220), (213), (114), (310), (321), (224), (314), and (206) tetragonal phases, respectively.^17,24^ These distinct visible peaks indicate high crystallinity of the spay-coated MAPI layer. However, the peaks located at 26.3° and 34.5° are not ascribed to any MAPI tetragonal or cubic phase and may be attributed to the (−122) and (133) planes from the PbI_2_–DMF complex formed during preparation.^24^ The MAPI lattice constants were estimated to be *a* = 8.83 Å and *c* = 12.69 Å, using XRD measurements.

The RT absorption spectra of the MAPI and p-GaN samples is shown in Fig. 1c. A sharp absorption edge and uniform band edge of p-GaN can be seen at 3.43 eV (361 nm), corresponding to band-to-band transitions, indicating very high crystal quality.^23^ The absorption edge observed at 1.56 eV is correlated to the MAPI bandgap.^17^ At the lower energy range, we observe a slight absorption band tail located below 1.56 eV, indicating a low concentration of shallow defects and high optical quality.^25^ These absorption measurements indicate that the MAPI/p-GaN PD can respond to a wide spectral range, spanning from UV to visible (800 nm) wavelength range.

To study the material optical quality, PL measurements were carried out at RT. Fig. 1d shows the PL spectrum of GaN, where a sharp peak is observed at 3.42 eV (362 nm), representing the band-edge emission, whereas the low energy shoulder at 3.28 eV is attributed to the donor–acceptor recombination from the conduction band (or shallow donors) to the doped Mg acceptor levels in the bandgap.^22^ Fabry–Perot fringes observed in the low energy shoulder are due to the GaN/air or substrate/GaN interface. No defect yellow luminescence (YL) band is observed, indicating superior crystal quality.^23,26^ The RT PL emission spectrum of MAPI film is shown in Fig. 1d. A dominant PL emission peak (red curve) is observed at 1.60 eV (775 nm) with a full width at half maximum (FWHM) value of ∼95 meV (47 nm), indicating high optical quality.^27^ This peak is due to the near band-to-band recombination.^17,27^

**Fig. 2:**
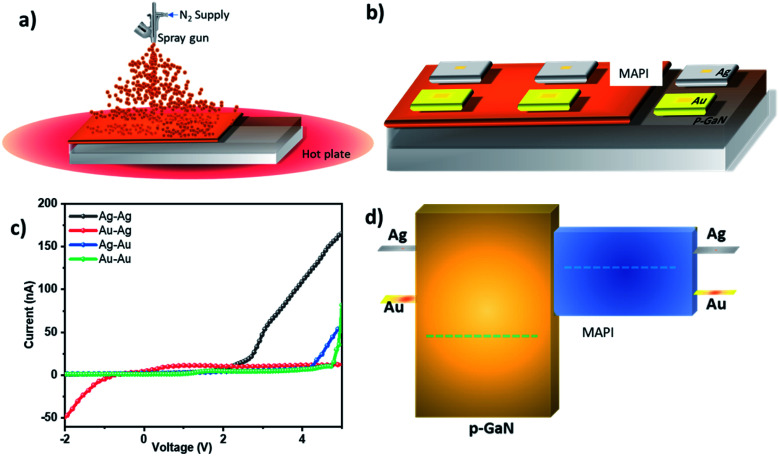
(a) A schematic of MAPI deposition by spray coating on GaN substrate; (b) the electrode configuration used in the MAPI/p-GaN device; (c) the current–voltage (*I*–*V*) characteristics of the asymmetric and symmetric electrode configurations in dark; and (d) the band diagram of the device with different electrodes.

Fig. 2a shows a schematic of MAPI deposition on p-GaN layer by spray-coating method, while different electrode combinations used in the devices are depicted in Fig. 2b. Au and Ag electrodes were deposited by e-beam deposition and four electrode combinations were investigated systematically to identify the most optimal contact configuration for enhancing photocarrier collection efficiency, namely: (a) symmetric configurations where both contacts are provided by either Au–Au configuration (*i.e.* Au/MAPI/p-GaN/Au) or Ag–Ag configuration (*i.e.* Ag/MAPI/p-GaN/Ag) and (b) asymmetric configurations, where Au–Ag configuration represents Au/MAPI/p-GaN/Ag and Ag–Au configuration denotes Ag/MAPI/p-GaN/Au. Our findings indicate that, when Ag electrodes are directly deposited on the MAPI layer, the material starts degrading, causing electrode damage. Such poor long-term stability of Ag–MAPI based devices can be a result of Ag electrode corrosion,^28^ while iodine migrates from the perovskite in the presence of Ag contacts. This adverse effect can be significantly limited by adopting Au contacts.^29^ Thus, we found that Au electrodes are the most stable contacts for MAPI as this metal contact improves both material and contact stability.

To explore the electric performance of the photodetector, current–voltage (*I*–*V*) measurements in dark were conducted for each electrode configuration. The *I*–*V* curves for symmetric (Au–Au, Ag–Ag) and asymmetric (Au–Ag and Ag–Au) configurations shown in Fig. 2c indicate that the dark current is the lowest in Au–Ag and Au–Au configurations, which in turn enhances photodetector sensitivity. In Au–Au and Ag–Au configurations, under light illumination, we also notice a rectifying behavior around 4–5 V range. Conversely, the highest dark current is attained in Ag–Ag electrode configuration, while Ag–Au configuration exhibits a higher dark current compared to that measured when using Au–Au.

**Fig. 3:**
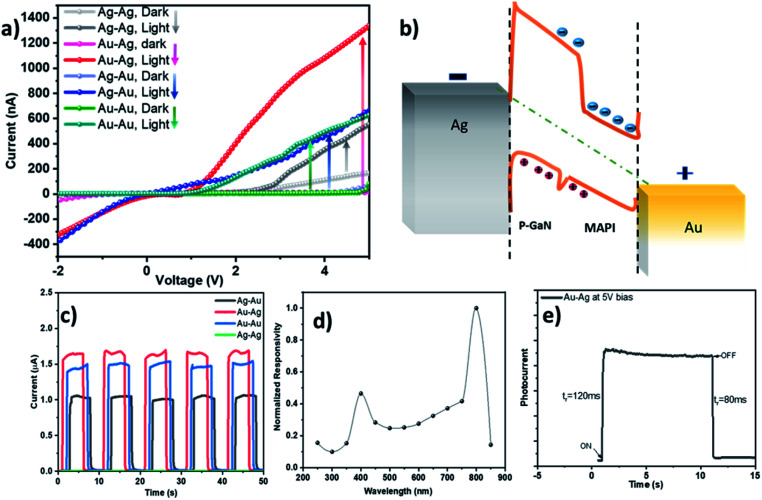
(a) *I*–*V* characteristics of the asymmetric and symmetric electrode configurations in dark and under illumination. (b) Schematic of the band alignment of the optimized Au/MAPI/p-GaN/Ag device in equilibrium and under bias condition. (c) The transient photocurrent (*I*–*T*) characteristics of the PD for different device configurations at 5 V and 40 mW cm^−2^ light power density; (d) wavelength dependence of the PD responsivity; and (e) rise and decay time of the device, using Au–Ag configuration, under white light illumination.

Fig. 2d shows a schematic of a scaled device band alignment. The Fermi level of p-GaN and MAPI has been calculated by Kelvin probe measurement. Fig. 3a shows the *I*–*V* characteristics obtained in dark as well as under illumination. To prevent MAPI degradation due to UV exposure, we designed our devices to allow the incident light (provided by a 40 mW cm^−2^ white lamp) to pass through GaN onto the MAPI layer, thus resulting in the maximum absorption in the UV range as well. It can be seen that the Au–Ag configuration yields maximum photocurrent, whereas Ag–Ag one exhibits minimum photocurrent.

To understand the effect of the symmetric and asymmetric contact configuration, we studied the band alignment of the device layers. It should be noted that, when the Au/MAPI is connected to positive bias and Ag/p-GaN is connected to negative bias, maximum photogeneration occurs. Therefore, in the subsequent discussions, this configuration is considered the device bias, as shown in Fig. 3b (presenting the proposed working principle of the device under this bias). In this bias, the potential difference due to the asymmetric electrode configuration assists the collection of photogenerated carriers at the electrodes. Thus, the photogenerated electrons are collected at the Au side, whereas the holes are collected at the Ag side. As the p-GaN Fermi level remains slightly away from that of Ag, we expect Schottky barrier generation in the Ag side, which possibly contributes to the rectifying behavior in the *I*–*V* characteristics of Au–Ag shown in Fig. 3a. In the opposite bias, where Ag is connected to positive and Au to negative bias, less photocurrent is generated as potential difference between the electrodes is reduced, resulting in insufficient low carrier collection. For the symmetric Au–Au and Ag–Ag electrode configurations, in the absence of potential difference, the Schottky barrier cannot be overcome, due to which a much lower photocurrent is measured relative to the Au–Ag configuration.

Transient photocurrent measurements were carried out for each configuration. Fig. 3c shows the photoresponse *I*–*T* characteristics (five on/off cycle of Ag–Ag is not obvious in the figure, as its photocurrent is in the nA range due to MAPI degradation as a result of Ag contact). It can be clearly observed that the device with the Au–Ag electrode configuration exhibits superior response, relative to that obtained for the Au–Au and Ag–Au configurations, confirming our hypothesis. Fig. 3d shows normalized responsivity (*R*) in the UV and visible spectral regions as one of the important PD parameters. *R* was measured, as it is an indication of the PD response efficiency to a light signal and is defined as the ratio of the generated photocurrent to the incident light intensity, *R* = Δ*J*_ph_/*SL*_light_, where Δ*J*_ph_ denotes the difference between the photocurrent and the dark current, *S* is the illuminated area, and *L*_light_ is the incident light density. It is evident that the photogeneration plateaus in the visible spectrum, with the exception of two peaks. These peaks are in close proximity to the GaN and MAPI bandgap edges, as confirmed by the absorption measurements shown in Fig. 1d. Therefore, photogeneration efficiency at the band edge of the heterojunction structure remains higher. For the optimized Au–Ag configuration, *R* = ∼55 mA W^−1^ at 5 V, under white light illumination (0.5 mW cm^−2^), which is the best value reported for MAPI/p-GaN PDs.^31^

The rise time (*t*_r_) and the fall time (*t*_f_) were calculated by considering *t*_r_ as the time difference between 10% and 90% of the highest photocurrent value when the photodetector is switched on, whereas *t*_f_ represents the time difference between 90% of the highest photocurrent and 10% of its value.^30^ The *t*_r_ is 120 ms and *t*_f_ remains below 80 ms, as shown in Fig. 3e, (both values are limited by the Keithley Source Meter 2400), demonstrating that the optimized contact configuration plays a very significant role in improving the GaN/MAPI device speed, which is three orders of magnitude faster than PVK/GaN PD reported in literature.^31^ These *I*–*T* findings demonstrate a fast response characteristic switching time for our p-GaN/MAPI photodetector compared to hybrid perovskite-inorganic semiconductor-based photodetector devices produced in previous studies.^13,17,32–34^

## Conclusions

In this work, we demonstrated that engineering the band alignment by using asymmetric electrode configurations (Au/MAPI/p-GaN/Ag) can significantly enhance photodetector performance. The most promising emerging perovskite material was combined with the highly stable and UV sensitive GaN material to produce an enhanced performance broadband MAPI/p-GaN PDs. The UV sensitivity of the device is favorable and the typical perovskite degradation due to direct exposure to UV radiation was eliminated by illuminating the device from the GaN side. These findings demonstrate the great potential of the III-nitride/MAPI-based PDs with high stability that are simple and economical to produce, while yielding wide spectral response required for a wide range of applications.

## Conflicts of interest

The authors declare no conflict of interest.

## Supplementary Material

RA-010-C9RA08823G-s001
