# Supplementary material for: Optimized performance III-nitride-perovskite-based heterojunction photodetector via asymmetric electrode configuration
Source: RSC Adv. 2020 Feb 11;10(10):6092–7. doi: 10.1039/c9ra08823g (PMC9049596; doi:10.1039/c9ra08823g)
Supplement: RA-010-C9RA08823G-s001 [file RA-010-C9RA08823G-s001.pdf]

Electronic Supplementary Material (ESI) for RSC Advances.
This journal is © The Royal Society of Chemistry 2020

Supporting Information

Optimized Performance III-Nitride-Perovskite-Based Heterojunction Photodetector via Asymmetric Electrode Configuration

Somak Mitra[a], Mufasila Mumthaz Muhammed[a], Norah Alwadai[ab], Dhaifallah.R. Almalawi[a], Bin Xin[a], Yusin Pak[a], , Iman S. Roqan[a]*

[a.]King Abdullah University of Science and Technology (KAUST), Physical Sciences and Engineering Division, Thuwal 23955-6900, Saudi Arabia.

[b.]Department of Physics, Princess Nourah bint Abdulrahman University (PNU), Riyadh 11671, Saudi Arabia.

Section 1: SEM cross section image

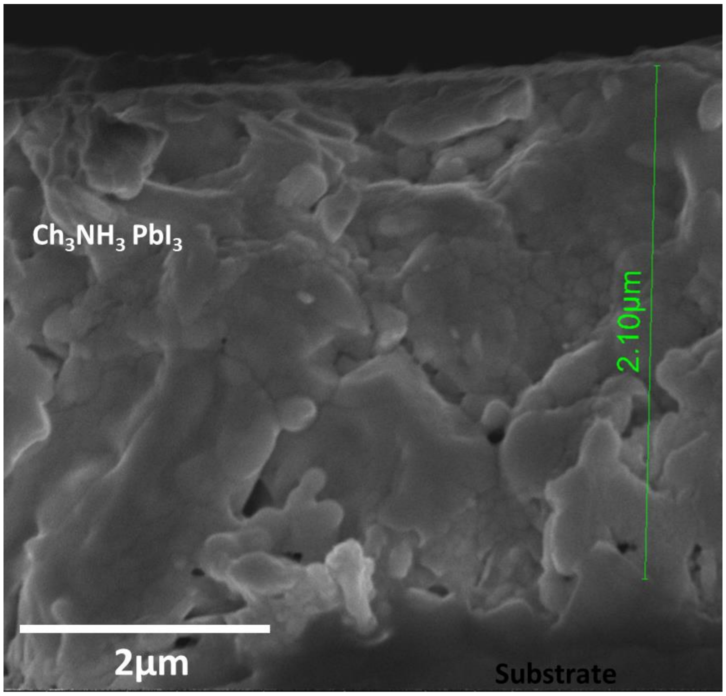

Figure S1: SEM cross-section image of the $CH_3NH_3PbI_3$ film spray coated in Al sheet.
